# Supplementary material for: DNA damage drives antigen diversification in Trypanosoma brucei
Source: Nature. 2026 Apr 8;654(8117):219–28. doi: 10.1038/s41586-026-10337-6 (PMC13233330; doi:10.1038/s41586-026-10337-6)
Supplement: Supplementary file 1 — Supplementary Methods, Table 1, Figs. 1–3 and references. [file 41586_2026_10337_MOESM1_ESM.pdf]

nature portfolio

https://doi.org/10.1038/s41586-026-10337-6

Supplementary information

DNA damage drives antigen diversification in *Trypanosoma brucei*

In the format provided by the authors and unedited

Supplementary Methods

Additional data availability details:

Sequences from assembled VSGs expressed by isolated parasite clones and colonies, raw FASTQ reads from parasite colonies (Smith2026: nanopore_colony_consensus_builder), donor VSG amplicons with corresponding mosaic VSG sequences, and parental mosaic colony VSG sequences (Smith2026: donor_intact_assay) can be found at github.com/mugnierlab/Smith2026 and on Zenodo (https://doi.org/10.5281/zenodo.18716076)[2].

Data from the output of VSG-AMP-seq can be found within github.com/mugnierlab/Smith2026/tree/main/figures and are associated with each corresponding figure.

VSG-seq from *in vivo* mouse infections (blood & tissues) is from Beaver et al[3]. and can be found at github.com/mugnierlab/Beaver2022.

VSG sequences were obtained from tryps.rockefeller.edu/Seqeuences.html[4]. Additional VSG sequences were identified from TriTrypDB[5,6] release 66 of the Lister2018 genome[7].

https://tritrypdb.org/a/service/raw-files/release-66/TbruceiLister427_2018/gff/data/TriTrypDB-66_TbruceiLister427_2018.gff

https://tritrypdb.org/a/service/raw-files/release-66/TbruceiLister427_2018/fasta/data/TriTrypDB-66_TbruceiLister427_2018_Genome.fasta

VSG N-terminal domain HMM profiles can be found at github.com/mugnierlab/find_VSG_Ndomains.

Plasmids:

pLEW100v5-BSD-FLAG-La-Cas9 was synthesized from pLEW100v5-BSD and pRPaCas9[8]. A *T. brucei* codon optimized FLAG tag was added to the N-terminus of Cas9. pLEW100V5-BSD was a gift from George Cross (Addgene plasmid #27658; http://n2t.net/addgene:27658; RRID:Addgene_27658). pRPaCas9 was a gift from David Horn (Addgene plasmid #111819; http://n2t.net/addgene:111819; RRID:Addgene_111819).

pUC19-HYG-BES1-AnTat1.1-telo and pUC19-HYG-BES1-VSG-228-telo were synthesized from pUC19-HYG-BES1-VSG-3-S317A-telo. This was a gift from Joey Verdi. AnTat1.1 sequence was obtained from AnTat1.1 specific cDNA from mouse infection D6 cloned into a pMiniT vector with the PCR Cloning Kit (NEB, E1202S). VSG-228 was partially amplified from VSG PCR (see below) derived from AnTat1.1 depleted D16 µMT parasites from blood via MACS[9]. The partial fragment was amplified with an AnTat1.1 family specific forward primer (5' -ACTACACCCACAACAAGCTCTA-3') and a pan VSG reverse primer which binds to a conserved 14bp region of the 3' UTR (5'-

GATTTAGGTGACACTATAGTGTTAAAATATATC-3') with AmpliTaq Gold (Applied Biosystems, 4398881) (anneal & extension 60C 1m, 35 cycles).The resulting PCR was cloned into the pMiniT backbone and the remainder of VSG-228 was *de novo* synthesized with Gibson Assembly.

The pT7sgRNA plasmids were obtained according to Rico et al.[8] pT7sgRNA was a gift from David Horn. (Addgene plasmid #111820; http://n2t.net/addgene:111820; RRID:Addgene_111820)

pHD309-PUR-VSG-228 was synthesized from pHD309-HYG-PUR. To obtain a plasmid for VSG-228 insertion, the sequence encoding the puromycin resistance gene was replaced with VSG-228. To convert the plasmid from hygromycin resistance to puromycin resistance, the sequence encoding the hygromycin resistance gene was replaced by the puromycin resistance gene. pHD309-HYG-PUR was a gift from George Cross. (Addgene plasmid #24014; http://n2t.net/addgene:24014; RRID:Addgene_24014)

pLEW100v5-PUR-VSG-228 was synthesized from pLEW100v5-BSD. The sequence encoding the blasticidin resistance gene was replaced with puromycin resistance. The sequence encoding the firefly luciferase was replaced with VSG-228. The rRNA promoter and the Tet operator sequences upstream of VSG-228 were removed.

pLEW100v5-177-PUR-VSG-228 was synthesized from pLEW100v5-177-HYG. The sequence encoding the hygromycin resistance gene was replaced with puromycin resistance. The sequence encoding firefly luciferase was replaced with VSG-228. The Tet operator sequences were removed. pLEW100v5-177-HYG was a gift from George Cross. (Addgene plasmid # 24013; http://n2t.net/addgene:24013; RRID:Addgene_24013)

pLEW100v5-PUR-T7-sgRNA was synthesized from pLEW100v5-PUR-VSG-228 and pT7sgRNA. 5' UTRs for Fructose 1,6-bisphosphate aldolase replaced the Actin 5' UTR for puromycin expression. Actin and GPEET 5'UTRs were removed as they have BbsI restriction sites within them. A T7 promoter was added to drive PURO expression. The T7-sgRNA cassette from pT7sgRNA was inserted into the modified pLEW backbone. Target specific constructs were obtained as above for pT7-sgRNA.

pLEW100v5-PUR-VSG-228_{noUTRs}, pLEW100v5-PUR-VSG-228_{500bp}, pLEW100v5-PUR-VSG-228_{400bp}, pLEW100v5-PUR-VSG-228_{300bp}, pLEW100v5-PUR-VSG-228_{200bp}, pLEW100v5-PUR-VSG-228_{100bp}, and pLEW100v5-PUR-VSG-$228_{100bpoffset}$ were synthesized from pLEW100v5-BSD. The sequence encoding the blasticidin resistance gene was replaced with puromycin resistance. The sequence encoding the firefly luciferase was replaced with a truncated versions of VSG-228 from pLEW-PUR-VSG-228 such that the UTRs were removed and then the coding sequence was successively trimmed down around the position within VSG-228 which corresponds to the AnTat1.1 694 cut site.

pLEW100v5-BSD-VSG-228 was synthesized from pLEW100v5-PUR-VSG-228. The puromycin resistance gene was replaced with blasticidin resistance.

pLEW100v5-NEO-T7-sgRNA was synthesized from pLEW100v5-PUR-T7-sgRNA and pLEW13. The puromycin resistance gene was replaced with the neomycin resistance gene from pLEW13. pLEW13 was a gift from George Cross. (Addgene plasmid # 24007; http://n2t.net/addgene:24007; RRID:Addgene_24007)

pJ1339 originated from Dr. Jack Sunter, Oxford Brookes University and has been referenced here[10,11].

The plasmids used to knockout RAD51(Tb927.11.8190), pJM-RMC-01 and pJM-RMC-03, were synthesized by swapping the UTRs of RAD51 into pyrFEKO-PUR and pyrFEKO-BSD using restriction enzyme cloning. pyrFEKO-PUR and pyrFEKO-BSD were gifts from George Cross[12]. (pyrFEKO-PUR: Addgene plasmid # 24021; http://n2t.net/addgene:24021; RRID:Addgene_24021) (pyrFEKO-BSD: Addgene plasmid # 24024; http://n2t.net/addgene:24024; RRID: Addgene_24024).

Three additional plasmids were utilized for knocking out RAD51 and BRCA2. pLEW100cre was a gift from John Donelson via George Cross. The sequence is here: tryps.rockefeller.edu/Plasmids/pLEW100cre.txt[12,13]. pPOTv7-blast-mNG was a gift from Samuel Dean and Keith Gull via Eva Gluenz. (Addgene plasmid # 179817; http://n2t.net/addgene:179817; RRID:Addgene_179817)[14,15] pPOTv7-g418-mNG was a gift from Samuel Dean and Keith Gull via Eva Gluenz. (Addgene plasmid # 179821; http://n2t.net/addgene:179821; RRID:Addgene_179821)[14,15]

Transgenic parasites:

To obtain the following transgenic parasites, 5 million parasites were electroporated with 5-10ug of digested plasmid with an AMAXA Nucleofector II using X-001 in Human T-cell Nucleofector Solution (Lonza VPA-1002). All parasites were maintained in selection unless otherwise specified. Detailed descriptions of the generation of transgenic parasites can be found in the Supplementary Methods.

For tetracycline-inducible Cas9 parasites, EATRO1125 or Lister427 parasites were electroporated with pLEW100v5-BSD-FLAG-La-Cas9, digested with NotI-HF (NEB, R3189S). Parasites were immediately selected in 5ug/mL blasticidin (Thermo Scientific, R21001).

To obtain AnTat1.1 and VSG-228 expressing Lister427 parasites, Lister427 parasites were electroporated with pUC19-HYG-VSG plasmids digested with BamHI-HF (NEB, R3136S). After 16-24 hours recovery, 25ug/mL of hygromycin (Fisher Scientific, J67371-XF) was added to the culture. After obtaining colonies 5-7 days later, selection was reduced to 5ug/mL.

Lister427 parasites with pLEW100v5-BSD-FLAG-La-Cas9 inserted were additionally electroporated with pUC19-HYG-VSG plasmid to express Antat1.1. Clones were obtained as above.

To obtain AnTat1.1 tetracycline-inducible Cas9 Lister427 parasites with a silent VSG-228 donor inserted, these parasites were additionally electroporated with pHD309-PUR-VSG-228, pLEW100v5-PURO-VSG-228 or pLEW100v5-177-PURO-VSG-228 plasmids digested with NotI-HF. After 16-24 hours of recovery, 0.1ug/mL of puromycin (Millipore Sigma, P8833) was added to the culture. Parasites were maintained in puromycin, and 5ug/mL of blasticidin and 5ug/mL hygromycin.

To obtain AnTat1.1 Lister427 parasites with silent, truncated fragments of the VSG-228 donor, AnTat1.1 expressing, tetracycline-inducible Cas9 Lister427 parasites were electroporated with pLEW100v5-PUR-VSG-228_{noUTRs}, pLEW100v5-PUR-VSG-228_{500bp}, pLEW100v5-PUR-VSG-228_{400bp}, pLEW100v5-PUR-VSG-228_{300bp}, pLEW100v5-PUR-VSG-228_{200bp}, pLEW100v5-PUR-VSG-228_{100bp}, or pLEW100v5-PUR-VSG-$228_{100bpoffset}$ plasmids digested with NotI-HF. After 16-24 hours recovery, 0.1ug/mL of puromycin was added to the culture. Parasites were maintained in puromycin, 5ug/mL blasticidin and 5ug/mL hygromycin.

Genomic DNA was obtained for these parasites to confirm insertion of the VSG-228 plasmid with the correct size. Parasites were harvested via centrifugation at 2600xg for 4 mins. Parasites were washed once with ~500uL of PBS, spun at 2600xg for 4 mins, the PBS was removed, and the pellet of cells was snap frozen in liquid nitrogen. DNA was extracted using the Monarch Spin gDNA Extraction Kit (NEB, T3010S). Verification was performed via PCR of the pLEW plasmid (10-40 ng of input gDNA; Fwd: 5'-CTGTGCCCCCGGTACGG-3'; Rev: 5'-CAAACCGACTCTGACGGCAG-3', no UTRs: annealing temp 55C, extension 2m 30s, 500-100bp: annealing temp 55C, extension 45s) for 35 cycles using OneTaq DNA Polymerase (NEB, M0480L).

To obtain constitutive guide expressing parasite lines, inducible Cas9-expressing parasites (both Lister427 and EATRO1125 Cas9 strains) were electroporated with pT7sgRNA guide containing plasmids digested with NotI-HF[8]. After 16-24 hours recovery, single colonies of parasites were selected with 2ug/mL phleomycin (Sigma-Aldrich, SML3001). Parasites were maintained without phleomycin selection. These parasites were used to obtain parasite clones following DNA break induction.

To obtain constitutive guide expressing parasite lines with puromycin selection rather than phleomycin, EATRO1125 inducible Cas9-expressing parasites were electroporated with pLEW-T7-sgRNA digested with NotI-HF. After 16-24 hours of recovery single colonies were selected with 0.1ug/mL of puromycin. Cultures were maintained in 0.1ug/mL puromycin and 5ug/mL blasticidin. These parasites were used for western blot analysis, death assays, and to obtain parasite clones following DNA break induction.

To obtain Cas9, mosaic AnTat1.1 Lister427 parasites with a silent VSG-228 donor and constitutively expressed guide, Single Marker 427 1339 parasites maintained in 0.1ug/mL puromycin were electroporated with pUC19-HYG-BES1-AnTat1.1-telo digested with BamHI-HF. After 16-24 hours recovery, 5ug/mL hygromycin was added to the culture to obtain clones. Upon visual identification of a clone in a 96-well plate, clones were challenged with 25ug/mL hygromycin for 24 hours and the VSG sequence of surviving

clones was validated with by VSG amplicon sequencing performed by Plasmidsaurus using Oxford Nanopore Technology with their custom analysis and annotation to determine those which expressed AnTat1.1. Following conformation of AnTat1.1 expression, parasites were maintained in puromycin and 5ug/mL hygromycin. Parasites were then electroporated with pLEW100v5-BSD-VSG-228 digested with NotI-HF. Following 16-24 hours of recovery, 5ug/mL blasticidin was added to the culture. These cultures grew slowly. Finally, parasites were electroporated with pLEW100v5-NEO-T7-sgRNA digested with NotI-HF. One set of parasites was treated immediately with 1ug/mL G418 (Sigma-Aldrich, G5013-250MG), while the remainder were treated with 1-3 ug/mL G418 following 16-24 hours of recovery. Additionally, AnTat1.1 Single Marker 427 1339 parasites were electroporated with pLEW100v5-NEO-T7-sgRNA digested with NotI-HF. Clones were treated after 16-24 hours recovery with 1ug/mL G418.

To obtain the following transgenic parasites, 20 million parasites were electroporated with 10 ug of digested plasmid unless otherwise specified with an AMAXA Nucleofector II using Z-001 in Tb transfection buffer[16]. These parasites were also cultured in HMI-11 with a maximum concentration of $1*10^6$ parasites/mL. Proper gene knockout was assessed in surviving clones by qRT-PCR.

To obtain EATRO1125 + J1339 ΔRAD51, EATRO1125 AnTat1.1 J1339 pleiomorphic parasites were electroporated with pJM-RMC-03 digested with PvuII-HF (NEB UK, R3151S) and XhoI (NEB UK, R0146S). These were plated immediately after transfection in three 24-well plates (at 1:20, 1:50 and 1:100) and the remaining parasites were retained in a flask. After 16 hours recovery, 2.5 ug/mL blasticidin (Invivogen, ant-bl-1) was used for selection. The second allele was knocked out using the LeishGEdit protocol[17] with the selections: Knockout, pPOT plasmids, and *Trypanosoma brucei* Lister strain 427. Sequences were checked against the EATRO1125 genome from TriTrypDB[5,6]. The donor primers were moved 80-100 bp upstream of the guide. To obtain the parasites, surviving parasites that were blasticidin resistance positive by PCR were electroporated with a 10ug pool of the following fragments: 1) a pPOTv7-g418-mNG amplicon synthesized with Phusion polymerase (NEB UK, M0530S) (Rad51 LeishGEdit donor primers, annealing temp 65C, extension 2m 15s). 2) T7-promoter driven forward and reverse guides flanking RAD51 synthesized with Phusion Polymerase (NEB UK, M0530S) (Rad51 LeishGEdit guide primers paired with G00, annealing temp 60C, extension 15s) Fragments were purified with ethanol precipitation. These parasites were immediately plated out in three 24-well plates (at 1:10, 1:50, and 1:100) and selected with 1 ug/mL G418 (Invivogen, ant-gn-1) and 2.5 ug/mL blasticidin after 16 hours of recovery.

To obtain EATRO1125 + J1339 ΔBRCA2, EATRO1125 AnTat1.1 J1339 pleiomorphic parasites were electroporated with a 10ug pool of the following fragments. 1) a pPOTv7-blast-mNG amplicon synthesized with Phusion polymerase (Brca2 LeishGEdit donor primers moved ~100 bp upstream of the guide, annealing temp 65C, extension 2m 15s). 2) T7-promoter driven forward and reverse guides flanking BRCA2 synthesized with Phusion polymerase (Brca2 LeishGEdit guide primers paired with G00, annealing temp 60C, extension 15s). Fragments were purified with ethanol precipitation. These parasites were immediately plated in two 24-well plates (at 1:20 and 1:60) with the

remaining parasites retained in a flask and selected with 2.5 ug/mL blasticidin (Invivogen, ant-bl-1) after 16 hours of recovery. Surviving parasites were checked by PCR and a second round of allele targeting was repeated as above except using pPOTv7-g418-mNG as the template for the amplicon. Clones were immediately plated in two 24-well plates (at 1:5) with the remaining parasites retained in a flask and selected with both 1ug/mL G418 (Invivogen, ant-gn-1) and 2.5 ug/mL blasticidin after 16 hours of recovery.

To obtain EATRO1125 ∆RAD51, EATRO1125 90-13 AnTat1.1 parasites were electroporated with pJM-RMC-03 digested with PvuII-HF (NEB UK, R3151S) and XhoI (NEB UK, R0146S). These parasites were immediately plated in two 24-well plates (at 1:20 and 1:60) with the remaining parasites retained in a flask and selected with 2.5 ug/mL blasticidin (Invivogen, ant-bl-1) after 16 hours. Surviving clones were electroporated with pJM-RMC-01 digested with PvuII-HF and XhoI. These parasites were selected with 0.2ug/mL puromycin and 2.5ug/mL blasticidin and plated as before. To remove the selectable markers, pLEW100cre was transiently electroporated into parasites and cre-recombinase activity was induced with 1ug/mL tetracycline (Sigma-Aldrich, T7660) as per Scahill et al.[18] Parasites were cloned in two 24-well plates (at 1:50 and 1:100) and selected using 75 ug/mL ganciclovir (Sigma-Aldrich, G2536) 7 hours post transfection. Clones were checked for sensitivity to puromycin/blasticidin.

To obtain EATRO1125 ∆RAD51 + J1339 parasites, EATRO1125 ∆RAD51 parasites were electroporated with pJ1339 digested with HindIII-HF (NEB UK, R3104S) and selected with 0.2ug/mL puromycin after 16 hours recovery. The surviving pool after 7 days was cloned to <1 parasite per well of a 96-well plate. Proper Cas9 expression was assessed in surviving clones by qRT-PCR.

qPCR:

To assess the expression of Cas9 and the knockout of RAD51 and BRCA2, gene expression was assessed by qPCR. Briefly, RNA was isolated from cell lines using Qiagen RNeasy Mini Kit (Qiagen, 74104) and cDNA was synthesized using Superscript IV (Invitrogen, 18090050) with random primers following the manufacturer's instructions. qPCR was performed with the Fast SYBR Green Master Mix (Applied Biosystems, 4385612) according to the manufacturer's protocol. (40 cycles, 0.25 conc of primers, 4uL of a 1:10 dilution of cDNA) Plates were read with an Applied Biosystems 7500 Fast Real-Time PCR System.

VSG-AMP-seq development:

VSG-AMP-seq is a sensitive targeted amplicon sequencing approach to identify mosaic derivatives of a VSG of interest based upon CRISPR GUIDE-seq[1]. Libraries are prepared by fragmenting VSG-specific cDNA. Fragments are then end-repaired, A-tailed and ligated to universal adapters containing a 25bp UMI. A VSG target of interest is selected, and a series of staggered primers are designed to cover the length of the target VSG's coding sequence. By pairing target-specific primers with a universal reverse adapter primer, target VSG fragments are amplified within a sample regardless of their identity. Mosaic reads are defined as those for whom a portion of the read matches the target and the remainder matches another VSG (the "donor VSG") within the VSG

repertoire (VSGnome) of the strain being studied. Due to its selective, target-specific amplification, this method can sensitively detect thousands of rare diversification events.

To validate this approach, we mixed together at a known ratio parasite clones expressing two distinct VSGs that shared sufficient sequence similarity to make them prone to the generation of PCR chimeras. We then performed VSG-AMP-seq on RNA extracted from this parasite mixture. This analysis revealed very few erroneous recombination events, even without UMI consolidation, demonstrating that VSG-AMP-seq is highly accurate and detects true mosaic recombination events.

VSG-AMP-seq library preparation:

VSG-AMP-seq was based upon AMP-seq[19] and GUIDE-seq[1]. cDNA was synthesized from DNA-free RNA using Superscript III Reverse Transcriptase and a VSG-specific primer which binds to a conserved 14-bp sequence within the 3' UTR. (5'-GTGTTAAAATATATC-3'). Second Strand synthesis was performed with NEBNext mRNA Second Strand Synthesis Module (E6111L). The resulting double-stranded cDNA was purified with 1.8X Mag-Bind Total NGS Beads. cDNA was fragmented briefly with NEBNext dsDNA Fragmentase (M0348L) for ten minutes at 37C to obtain fragments of approximately 500bp. Fragments were purified with a double-sided Mag-bind bead cleanup. First, fragments were incubated with 0.5X beads, DNA bound to the beads were discarded and an equal volume of beads as before, then a 1X PEG concentration, was added to the fragments and the cleanup proceeded as normal. Fragments were end-repaired with Enzymatics low concentration end repair mix (Y9140-LC-L) and A-tailed with Recombinant Taq (Life Technologies, 100021276). Y-Adapters were pre-annealed by incubating a MiSeq Common Adapter with Adapters (A01-A10) containing 25 bp Unique Molecular Indexes (UMIs) and a barcode at 10uM at 95C for 1 sec, 60C for 1s, and slowly cooled to 4C. Adapters were ligated to the A-tailed fragments with T4 DNA Ligase (Enzymatics L6030-LC-L). The resulting fragments were cleaned up with 0.8X Mag-Bind beads. Two target specific PCRs were performed on the fragments with pools of target specific primers, one in the forward and one in the reverse direction. All primers are listed in Table 1. Target primers have spacers of varying lengths to generate sequence diversity without the need for PhiX. Target specific primers are paired with P5_2 and a sample specific P primer (P701-P710) which contains a second barcode. Fragments are amplified with Platinum Taq (Life Technologies, 10966018) using the following program: 95C 5 mins, 35 cycles of 95C for 30s, 55C for 30s, and 72C for 30s, and 72 for 5 mins. The resulting products were cleaned up with 0.6X-0.7X Mag-bind beads. Libraries were quantified using the Qubit dsDNA HS Kit (Life Technologies, Q32854) and run on a 1% agarose gel to determine average length. Libraries were sequenced on a MiSeq or for deep sequencing of μMT samples with wildtype parasites on a NovaSeq6000 with custom index1 and read2 primers using the following cycle conditions: "151|8|33|131" with the paired-end Nextera sequencing protocol. For deep sequencing of the constitutively expressed Cas9 EATRO1125 and corresponding knockout cells, an additional targeted library was prepared with 2F and 2R as the only primers. The typical library and the targeted library were each sequenced on a lane of the NovaSeq6000.

VSG-AMP-seq analysis:

Index sequences were added to the names of sequencing reads. FASTQ files were demultiplexed by target specific primer using cutadapt v=3.5[20] searching for multiple target-specific primers at the 5' end of read2 in paired-end mode using flags --action=retain --overlap 10. Then, reads were quality trimmed with trim_galore v0.6.4_dev (github.com/FelixKrueger/TrimGalore) where adapter sequences, if present, were removed with flags --dont_gzip --paired --trim1. Spacers were removed from read1 if present using cutadapt by searching for the target-specific primer sequence at the 3' end of the read with flags --action=retain --overlap 10. Dual-indexed barcodes were used to demultiplex reads. A custom function in the pipeline (barcode_errors()) was used to the identify number of mismatches permitted so all barcodes present could be unambiguously identified.

To consolidate reads, UMIs were extracted from large FASTQ files, split into smaller files and grouped by 100% identity using cd-hit-est v4.8.1[21,22] with flags -c 1.0 -n 8 -M 16000 -d 0. UMIs were then grouped by 92% (no more than 2 mismatches per UMI) using flags -c 0.92 -n 8 -M 16000 -d 0. Reads were sorted into consensus groups based on UMIs and a consensus sequence was formed as the most popular base at each position of the read with a threshold quality score of at least 2. If there was a tie, an N was used. Clusters with fewer than 3 reads were removed. Consolidation was only used for samples isolated from WT and muMT$^-$ mouse infections with wildtype parasites (consol_reads.py) and for the deep sequenced constitutively expressed Cas9 EATRO1125 and corresponding knockout cells (ΔRAD51, ΔBRCA2) (consol_reads_updated.py).

The full sequence of the AnTat1.1 transcript was determined by Plasmidsaurus using Oxford Nanopore Technology with their custom analysis and annotation. However, in our initial analyses of control samples, we determined that many reads slightly differed near the splice leader, suggesting that transcripts might be alternatively spliced or included extra sequence appended at some point during the library preparation process, specifically adjacent to splice leader. We defined the AnTat1.1 reference as the full-length variant determined by Plasmidsaurus sequencing, with the longest splice variant observed in control samples (no guide, electroporated) appended to the 5'-end. When reads did not align to this reference, they were then compared against the ten most commonly observed AnTat1.1 isoforms to account for all possible variations in the AnTat1.1 transcript. These sequences, including the full length AnTat1.1 reference, can be found in global_target.py. The positions were 0 indexed. Read2, the anchored read, was aligned to AnTat1.1. Read pairs were removed if: the first 4/10 bases after the primer did not align to AnTat1.1, an alternative VSG which contained the primer sequence was amplified, the wrong position of AnTat1.1 was amplified by the anchor, if the reads were too short (<15 bp), contained too many Ns (>5), if there was an inversion event or a duplication, or if the transcript was alternatively spliced. Read1, the unanchored read, was also aligned to AnTat1.1 with a mismatch of up to 1bp. If no alignment could be found, read1 was trimmed to remove AnTat1.1 like sequences from the 5' and 3' ends, allowing up to 1 mismatch on each end to be removed, leaving a fragment which can be used to search the VSGnome and identify potential donor VSGs. A consensus sequence was

generated from the reads, if they overlapped, using their alignment positions within AnTa1.1. AnTat1.1, the consensus read, and putative donor VSG were aligned, and recombination sites were identified. If no consensus could be generated from the two reads, read1 was used. Ambiguous recombination sites where the mosaic portion of a read matched more than one donor were represented by the average position of the potential recombination sites and identified as ambiguous. Mosaic reads which did not overlap to generate a consensus and did not contain an identifiable recombination site in read1 were excluded from analysis. A summary of these is found in supplemental excel file 3. The average proportion of mosaic VSGs without an identifiable recombination site was ~1.6% for *in vitro* samples with at least 10 mosaic reads identified (max = ~9%). Samples derived from *in vivo* mouse experiments tended to have more unidentifiable recombination sites, with a majority occurring in the 0R and 1R primer samples (avg = ~30%, max = ~43%).

To normalize the number of detected recombinations between Cas9 samples, read1 or consensus read1 was mapped to AnTat1.1 via bowtie(1.3.1) with the flags -v 2 -S –no-unal. The resulting SAM file was parsed to identify all primary alignments within 250 bp up and downstream from the from cut site. This count was used to normalize sequencing depth between samples.

The following additional software was used in the custom script for VSG-AMP-seq: Python(3.8.19), Biopython(1.78), Pandas(1.2.1), python-levenshtein(0.25.1), progress-bar(2.5), regex(2.5.82), and R(4.0.2).

VSG-seq analysis software:

VSG-seq(1)[23] was performed with the following programs: Trinity(2.8.5), Biopython(1.72), Blast(2.9), Bedtools(2.29.2), cd-hit(4.8.1), trim-galore(0.6.4), bowtie(v1.2.3), and samtools(1.9).

VSG clustering and family identification:

VSGs were identified from annotated genes and pseudogenes from the TriTrypDB-66 Lister427 2018 genome GFF[7]. Those identified with an attribute including “VSG” or “variant” but not “invariant”, “Invariant”, “histone”, “Histone”, “RING”, “ubiquitin”, ”exclusion”, “Exclusion”, “nonvariant”, or “Nonvariant”. Many known VSGs were only annotated as an unknown protein product. To identify additional VSG-encoding genes, we isolated all unknown gene products with attributes including “hypothetical protein”, “pseudogene” or “unknown”, but not including “VSG”, “variant” or any of the additionally excluded words above. VSGs were identified among these unknown genes via BLAST(2.10.0) against all known VSG-encoding sequences (Those from Cross et al.[4], the EATRO1125 VSGs with flanking sequences from George Cross, Beaver et al.[3], and TriTrypDB-66[5] TRU927, Lister427, and EATRO1125 genomes.) Genes were considered a VSG if the blast hit extended over 80% of the unknown gene and had a bitscore of 500 or greater. We identified an additional 2545 VSGs.

All Lister427 identified VSGs from the Lister427 2018 genome and the FASTAS from Cross et al.[4] (vsgs_tb427_all_atleast150aas_cds.txt,

vsgs_tb427_nodups_atleast250aas_cds.txt, vsgs_tb427_nodups_atleast250aas_cdsplusflanks.txt) were combined and duplicate VSGs were removed with cd-hit-est with the following flags: -c 1.0 -n 8 -M 16000 -d 0. VSGs with identical sequences, but distinct positions within the 2018 genome were added back for a total of 8564 VSGs. In the Lister427 2018 genome, we identified 5789 VSG-encoding genes total and included 300bp flanking sequences up and downstream of the coding sequence for further analysis. These are genes visualized in Supplemental Figure 4.

EATRO1125 VSGs were obtained from TrypsRU (George Cross) and included 200 bp flanking sequences. (vsgs_tb1125_all_atleast150aas_cdsplusflanks.txt) Duplicate genes were removed with cd-hit-est with the following flags: -c 1.0 -n 8 -M 16000 -d 0.

To define VSG families, network analysis was performed using the curated VSG reference FASTA files described above. Each genomic repertoire was subjected to an all-versus-all BLASTn run under the default parameters to generate pairwise tables which contained the query-subject pair, query sequence length, alignment length, E-value and percent identity. Networks were generated with the igraph R package[24,25] using undirected and unweighted clustering of nodes after applying link cutoffs based on E-value < 1e-20 and alignment coverage of the query sequence > 80%. The leading eigenvector clustering method (function: cluster_leading_eigen()) was used to detect communities and assign nodes based on their connectivity.

To supplement the BLAST network graph approach, the greedy clustering UCLUST algorithm[26] was used to assess clusters of VSGs. VSGs were clustered using usearch with the following flags: -id .75 -strand both -sizeout -sort length -maxhits 2. For each genome, identified VSGs were sorted by sequence length then clustered at a global identity threshold of 75%

Tb427VSG-8 and EATRO1125VSG-73 *in vitro* mosaic clonal analysis:

All but one population (VSG73_guide680_clone2) were not clonal, but rather mixed mosaic colonies. Two VSG-8 expressing parasites were subcloned to obtain clonal mosaic-expressing parasites. For the remaining colonies, consensus sequences for individual mosaics were made from the fragmented plasmidsaurus reads. Briefly, FASTQ files were converted to FASTA using sed (4.5) with the flag -n \'1~4s/^@/>/p;2~4p\'. Reads were then clustered with cd-hit-est(4.8.1) with the flags -c 0.99 -n 8 -M 16000 -T 16 -d 0. Clusters with 10 or more reads were mapped to the cluster leader with minimap2 (2.30-r1287)[27,28] with the flags -a –sam-hit-only -t 16, then using samtools (1.22.1)[29] the output SAM file was converted to BAM file, sorted, and converted to mpileup format. Using the mpileup format, a consensus sequence was generated such that if 85% of the clustering reads disagreed with the cluster leader sequence at a particular position, the base was modified. For indels, if 55% of the reads contained an indel, the consensus sequence was modified to reflect the majority sequence. Indels are common in nanopore sequencing. These consensus sequences aligned via BLAST[30] to the parental sequence and mosaic insertion was identified by eye. Consensus sequences and raw FASTQ files can be found at github.com/mugnierlab/Smith2026/tree/main/nanopore_colony_consensus_builder/.

Tb427VSG-8 mosaic detection *in vivo*:

Reads were basecalled with Dorado (7.2.13) using Super-accurate basecalling, 400bp. Resulting bam files were converted to FASTQ with samtools fastq (1.22.1)[29]. Reads were demultiplexed with cutadapt (4.9)[20] using flags: --revcomp -m 1000 --overlap 19 --action=lowercase -q 10 --cores 0. FASTQ files were converted to FASTA using sed (4.5) with the flag -n \'1~4s/^@/>/p;2~4p\'. Non-overlapping unique k-mers 20bp in length were identified for all reads. These were mapped to Tb427VSG-8 and VSG-4806 with hisat2 (2.1.0)[31]. k-mers which mapped to VSG-4806, but not Tb427VSG-8 were used for mosaic identification. All reads were tested and if 50 or more unique VSG-4806 k-mers were identified in a read, it was processed further as a putative mosaic. Putative mosaic VSGs were corrected by hand to remove indels, then mosaic recombination sites were identified from aligning VSG-8, VSG-4806, and the mosaic VSG. To normalize the number of reads per sample, all demultiplexed sequences between 1000 bp and 2000 bp were BLASTed(2.16.0+)[30] against the VSGs identified by George Cross[4] with the flags, -num_threads 16 -max_target_seqs 1 -max_hsps 1. Sequences with a BLAST hit longer than 200 bp were counted as true VSGs. Python scripts associated with this analysis can be found at github.com/Smith2026/tree/main/altVSG8_nanopore_analysis/.

Supplementary Table 1. Primer sequences

Guide Primers		
Primer Name	Sequence	
AnTat1.1_243	GAAATTAATACGACTCACTATAGGATTCAAAAACGGCCAAACGCCGTTTTAGAGCTAGAAATAGC	
AnTat1.1_369R	GAAATTAATACGACTCACTATAGGGGCGTAAATTAACAGTGTCGGTTTTAGAGCTAGAAATAGC	
AnTat1.1_694	GAAATTAATACGACTCACTATAGGAGTACAGACCCAGAAGCCAGGTTTTAGAGCTAGAAATAGC	
AnTat1.1_894	GAAATTAATACGACTCACTATAGGACGCCGGTGTCGCAGCTAAACGTTTTAGAGCTAGAAATAGC	
AnTat1.1_978R	GAAATTAATACGACTCACTATAGGGTCGTTGGCTGCTTGGAGTTGTTTTAGAGCTAGAAATAGC	
AnTat1.1_1459	GAAATTAATACGACTCACTATAGGACCAATCCAGAAAAGTGCAAGTTTTAGAGCTAGAAATAGC	
G00	AAAAGCACCGACTCGGTGCCACTTTTTCAAGTTGATAACGGACTAGCCTTATTTTAACTTGCTATTTCTAGCTCTAAAAC	
AnTat1.1_ upstream	GAAATTAATACGACTCACTATAGGGCAAAAAGGAGGAGAGGAAATGTTTTAGAGCTAGAAATAGC	
Annealed Guides for T7-sgRNA insertion		
Fragment Name	FWD	REV
AnTat1.1_243	AGGGATTCAAAAACGGCCAAACGCC	AAACGGCGTTTGGCCGTTTTTGAAT
AnTat1.1_369R	AGGGGGCGTAAATTAACAGTGTCG	AAACCGACACTGTTAATTTACGCC
AnTat1.1_694	AGGGAGTACAGACCCAGAAGCCAG	AAACCTGGCTTCTGGGTCTGTACT

AnTat1.1_894	AGGGACGCCGGTGTCGCAGCTAAAC	AAACGTTTAGCTGCGACACCGGCGT
AnTat1.1_978R	AGGGGTCGTTGGCTGCTTGGAGTT	AAACAACTCCAAGCAGCCAACGAC
AnTat1.1_1459	AGGGACCAATCCAGAAAAGTGCAA	AAACTTGCACTTTTCTGGATTGGT
Tb427VSG-2_707	AGGGACCAACGGCCTCGGCAAAAG	AAACCTTTTGCCGAGGCCGTTGGT
Tb427VSG-2_1082	AGGGCCAGTGGCGCAAAACCTGGT	AAACACCAGGTTTTGCGCCACTGG
Tb427VSG-8_783	AGGGGAACGGGCAAAACCCAAACG	AAACCGTTTGGGTTTTGCCCGTTC
Tb1125VSG-73_194	AGGGACTTGGTTGTTAGGACAGCGG	AAACCCGCTGTCCTAACAACCAAGT
Tb1125VSG-73_680	AGGGACTTGGGATGAAGAAGAGCTG	AAACCAGCTCTTCTTCATCCCAAGT
Tb1125VSG-73_1436	AGGGAAGATGGCTGTAAAGTGGAG	AAACCTCCACTTTACAGCCATCTT
DNase Verification Primers		
Target Name	FWD	REV
HSP-70 (Tb927.11.11330)	AGAACACTATCAATGACCCCAAC	CCATGCCCTGGTACATCT
HYG	ACAGCGGTCATTGACTGGAG	ATTTGTGTACGCCCGACAGT
pLEW VSG-228 Fragment Size Verification Primers		
Target Name	FWD	REV
pLEW Insertion Amplicon	CTGTGCCCCCGGTACGG	CAAACCGACTCTGACGGCAG
pLEW-Blasticidin gDNA Amplification		
Primer Name	FWD	REV
pLEW-Blast_gDNA	TTGACACCAGTGAAGATGCGG	CGGCAGTTTACGAGAGAGATGA
VSG-AMP-seq Primers		
Primer Name	Sequence	
All-VSG-3'UTR	GTGTTAAAATATATC	
Y-adapter%	[Phos]GATCGGAAGAGC*C*A	
A01^	AATGATACGGCGACCACCGAGATCTACACTAGATCGC(N:25252525)(N)(N)(N)(N)(N)(N)(N)(N)(N)(N)(N)(N)(N)(N)(N)(N)(N)(N)(N)(N)(N)(N)(N)(N)ACACTCTTTCCCTACACGACGCTCTTCCGATC*T	
A03^	AATGATACGGCGACCACCGAGATCTACACTATCCTCT(N:25252525)(N)(N)(N)(N)(N)(N)(N)(N)(N)(N)(N)(N)(N)(N)(N)(N)(N)(N)(	

	N)(N)(N)(N)(N)(N)ACACTCTTTCCCTACACGACGCTCTTCCGATC*T
A04^	AATGATACGGCGACCACCGAGATCTACACAGAGTAGA(N:25252525)(N)(N)(N)(N)(N)(N)(N)(N)(N)(N)(N)(N)(N)(N)(N)(N)(N)(N)(N)(N)(N)(N)(N)ACACTCTTTCCCTACACGACGCTCTTCCGATC*T
A05^	AATGATACGGCGACCACCGAGATCTACACGTAAGGAG(N:25252525)(N)(N)(N)(N)(N)(N)(N)(N)(N)(N)(N)(N)(N)(N)(N)(N)(N)(N)(N)(N)(N)(N)(N)ACACTCTTTCCCTACACGACGCTCTTCCGATC*T
A06^	AATGATACGGCGACCACCGAGATCTACACACTGCATA(N:25252525)(N)(N)(N)(N)(N)(N)(N)(N)(N)(N)(N)(N)(N)(N)(N)(N)(N)(N)(N)(N)(N)(N)(N)ACACTCTTTCCCTACACGACGCTCTTCCGATC*T
A07^	AATGATACGGCGACCACCGAGATCTACACAAGGAGTA(N:25252525)(N)(N)(N)(N)(N)(N)(N)(N)(N)(N)(N)(N)(N)(N)(N)(N)(N)(N)(N)(N)(N)(N)(N)ACACTCTTTCCCTACACGACGCTCTTCCGATC*T
A08^	AATGATACGGCGACCACCGAGATCTACACCTAAGCCT(N:25252525)(N)(N)(N)(N)(N)(N)(N)(N)(N)(N)(N)(N)(N)(N)(N)(N)(N)(N)(N)(N)(N)(N)(N)ACACTCTTTCCCTACACGACGCTCTTCCGATC*T
A09^	AATGATACGGCGACCACCGAGATCTACACGACATTGT(N:25252525)(N)(N)(N)(N)(N)(N)(N)(N)(N)(N)(N)(N)(N)(N)(N)(N)(N)(N)(N)(N)(N)(N)(N)ACACTCTTTCCCTACACGACGCTCTTCCGATC*T
A10^	AATGATACGGCGACCACCGAGATCTACACACTGATGG(N:25252525)(N)(N)(N)(N)(N)(N)(N)(N)(N)(N)(N)(N)(N)(N)(N)(N)(N)(N)(N)(N)(N)(N)(N)ACACTCTTTCCCTACACGACGCTCTTCCGATC*T
P5_2	AATGATACGGCGACCACCGAGATCTACAC
P701	CAAGCAGAAGACGGCATACGAGATTCGCCTTAGTGACTGGAGTCCTCTCTATGGGCAGTCGGTGA
P703	CAAGCAGAAGACGGCATACGAGATTTCTGCCTGTGACTGGAGTCCTCTCTATGGGCAGTCGGTGA
P704	CAAGCAGAAGACGGCATACGAGATGCTCAGGAGTGACTGGAGTCCTCTCTATGGGCAGTCGGTGA
P705	CAAGCAGAAGACGGCATACGAGATAGGAGTCCGTGACTGGAGTCCTCTCTATGGGCAGTCGGTGA
P706	CAAGCAGAAGACGGCATACGAGATCATGCCTAGTGACTGGAGTCCTCTCTATGGGCAGTCGGTGA
P707	CAAGCAGAAGACGGCATACGAGATGTAGAGAGGTGACTGGAGTCCTCTCTATGGGCAGTCGGTGA
P708	CAAGCAGAAGACGGCATACGAGATCCTCTCTGGTGACTGGAGTCCTCTCTATGGGCAGTCGGTGA
P709	CAAGCAGAAGACGGCATACGAGATATCACGGTGTGACTGGA

	GTCCTCTCTATGGGCAGTCGGTGA	
P710	CAAGCAGAAGACGGCATACGAGATCGATGTGCGTGACTGGA GTCCTCTCTATGGGCAGTCGGTGA	
AnTat1.1_1F[&]	CCTCTCTATGGGCAGTCGGTGAT(N)$_{0-7}$CGCAAACACTACAACGAGCC	
AnTat1.1_2F[&]	CCTCTCTATGGGCAGTCGGTGAT(N)$_{0-7}$CAGAATGCGACACGGAAAGC	
AnTat1.1_3F[&]	CCTCTCTATGGGCAGTCGGTGAT(N)$_{0-7}$ACGCAGGCGGCTTCAAAACA	
AnTat1.1_4F[&]	CCTCTCTATGGGCAGTCGGTGAT(N)$_{0-7}$AACAGCCGCAGCAACCAAAC	
AnTat1.1_0R[&]	CCTCTCTATGGGCAGTCGGTGAT(N)$_{0-7}$GGCCACAAATGCGGCAGAAAC	
AnTat1.1_1R[&]	CCTCTCTATGGGCAGTCGGTGAT(N)$_{0-7}$GCCATAAGCTGCGGTTTCGT	
AnTat1.1_2R[&]	CCTCTCTATGGGCAGTCGGTGAT(N)$_{0-7}$GTTGTGTATGGTTAGCAGGC	
AnTat1.1_3R[&]	CCTCTCTATGGGCAGTCGGTGAT(N)$_{0-7}$CTTGTATTTTGTGCGTGGCG	
Index1[%]	ATCACCGACTGCCCATAGAGAGGACTCCAGTCAC	
Read2[%]	GTGACTGGAGTCCTCTCTATGGGCAGTCGGTGAT	
VSG PCR Barcoding Primers		
14mer_B1_SP6	TACGATTTAGGTGACACTATAGTAGATCGCGTGTTAAAATAT ATC	
14mer_B2_SP6	TACGATTTAGGTGACACTATAGCTCTCTATGTGTTAAAATATA TC	
14mer_B3_SP6	TACGATTTAGGTGACACTATAGTATCCTCTGTGTTAAAATATA TC	
14mer_B4_SP6	TACGATTTAGGTGACACTATAGAGAGTAGAGTGTTAAAATAT ATC	
14mer_B5_SP6	TACGATTTAGGTGACACTATAGGTAAGGAGGTGTTAAAATAT ATC	
14mer_B6_SP6	TACGATTTAGGTGACACTATAGACTGCATAGTGTTAAAATATA TC	
14mer_B7_SP6	TACGATTTAGGTGACACTATAGAAGGAGTAGTGTTAAAATAT ATC	
14mer_B8_SP6	TACGATTTAGGTGACACTATAGCTAAGCCTGTGTTAAAATAT ATC	
14mer_B9_SP6	TACGATTTAGGTGACACTATAGGACATTGTGTGTTAAAATATA TC	
14mer_B10_SP6	TACGATTTAGGTGACACTATAGACTGATGGGTGTTAAAATAT ATC	
Knockout LeishGEdit Primers		
Target name	FWD	REV
RAD51 donor	GGTGAATCGTGGGGAATTAT	GGACAGAATAACGTGTACCA

(Tb927.11.8190)	TTGGGAGGTGGTATAATGCAGACCTGCTGC	TAAACACGACCCGGAACCACTACCAGAACC
RAD51 guide	GAAATTAATACGACTCACTATAGGCGCGTTAAGAAACCTTCACGGTTTTAGAGCTAGAAATAGC	GAAATTAATACGACTCACTATAGGATGACGAGGAAAAAAAGATGTTTTAGAGCTAGAAATAGC
BRCA2 donor (Tb927.1.640)	CCTCCACCTCCACACGCGTGCAATTGAATGGTATAATGCAGACCTGCTGC	CAGACTCTTGAGAGAACCTTGTCCGAACGCCCGGAACCACTACCAGAACC
BRCA2 guide	GAAATTAATACGACTCACTATAGGACACACGGGGAATTTCAGGAGTTTTAGAGCTAGAAATAGC	GAAATTAATACGACTCACTATAGGATGAAGGTATACCCGGCGAAGTTTTAGAGCTAGAAATAGC
qPCR Primers		
Target Name	FWD	REV
Cas9_1[32]	CCACTATGAGAAGCTGAAGGG	TTTGTCCAGATTAGCGTCGG
Cas9_2[32]	TACAACAAGCACCGGGATAAG	GTCGATGGTGGTGTCAAAGTA
RAD51 (Tb927.11.8190)	CCGTCCTGAGCGCTTGGTAG	GCGGACGCTTGCAACAACAA
BRCA2 (Tb927.1.640)	AGCACAGGTGGTGCGTTGAA	TTGGCACCGCAATCATCCCA
ZFP3[33] (Tb927.3.720)	CAGGGGAAACGCAAAACTAA	TGTCACCCCAACTGCATTCT
Miscellaneous Additional Primers		
Name	Sequence	
SL-FWD	ACAGTTTCTGTACTATATTG	
SP6-14mer-REV	GATTTAGGTGACACTATAGTGTTAAAATATATC	
AnTat1.1 Sanger Sequencing Primer	AGAGAATACTAAGCTAGTTGGC	
Pan AnTat1.1 family FWD	ACTACACCCACAACAAGCTCTA	
pJM-RMC Sanger Sequencing Primer	CGACCGAGCGCAGCGAGTCA	

[a] * = indicates a phosphorothioate bond modification
[b] [Phos] = 5’ phosphorylation
[c] ^ = hand mixing
[d] & = machine mixing
[e] % = HPLC purification

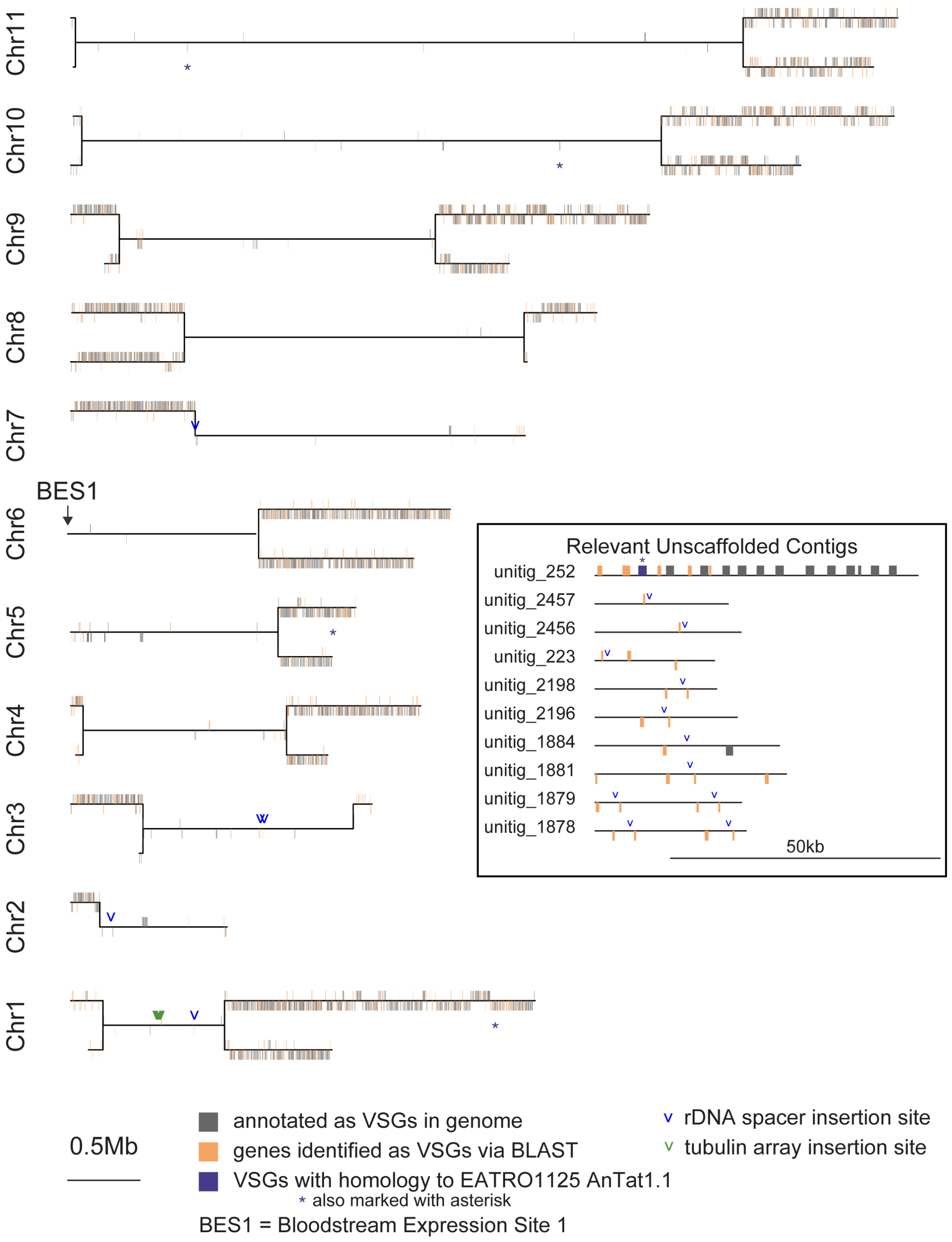

Supplementary Figure 1. The Lister427 VSG annotated genome
The megabase chromosomes from Müller et al.[7] VSGs annotated in the genome are plotted in gray. Unknown genes identified as VSGs via BLAST are plotted in yellow. Details of how these VSGs were identified are detailed in Supplemental Methods. AnTat1.1 homologous family members are colored purple and marked with an asterisk. Insertion sites for the VSG-228 are denoted by arrows at the insertion location. Inset are the unitigs which are unscaffolded and harbor a copy of the AnTat1.1 family member or a potential insertion site. Bloodstream Expression Site 1 (BES1) is on the 5' end of chromosome 6 and is marked by an arrow.

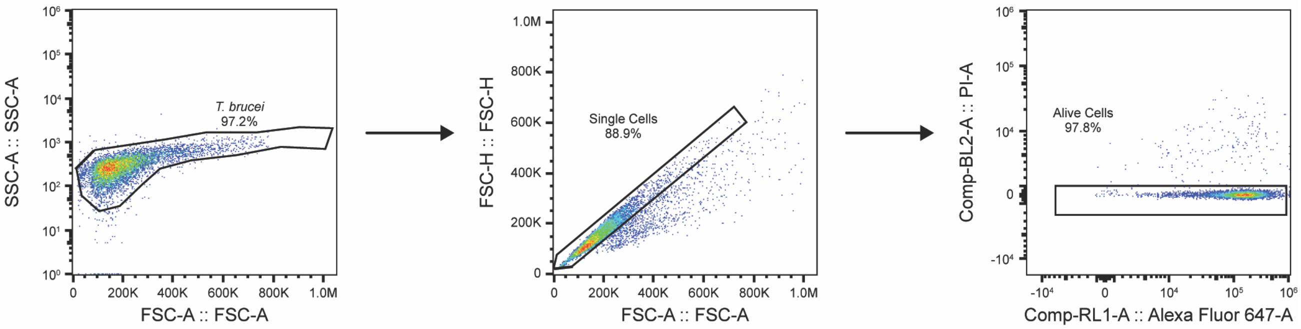

Supplementary Figure 2. Flow cytometry gating strategy for Fig. 5 & Extended Data Fig. 10

Example gating strategy for the flow cytometry experiment. Samples were first gated for typical *T. brucei* patterns, which removes some debris and dead cells. Then doublet discrimination was performed on samples to identify single cells. Finally, all alive cells were gated based upon negative PI staining, and this population was used to generate the histograms in Fig. 5 & Extended Data Fig. 10. This example is a Parental AnTat1.1 sample.

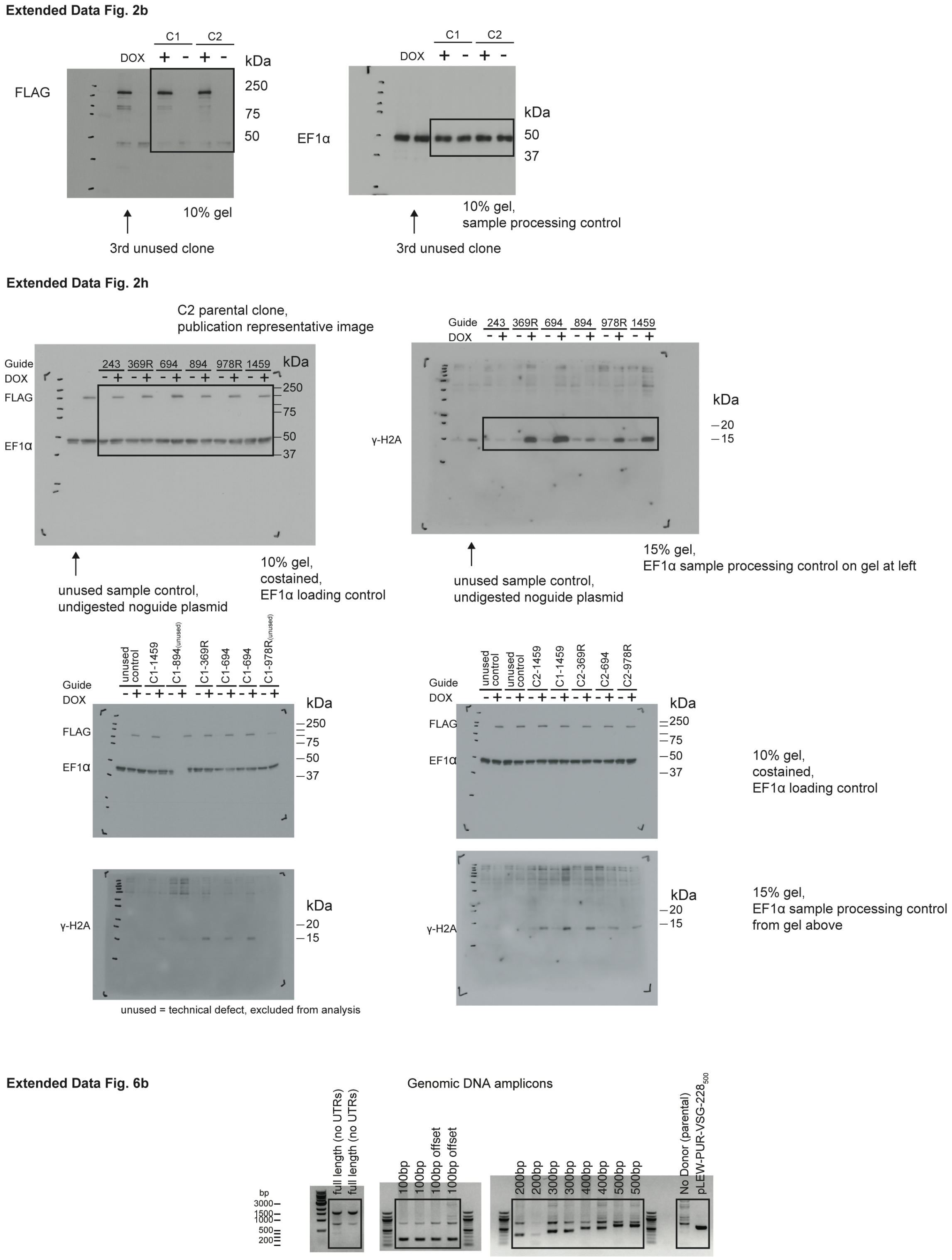

Supplementary Figure 3. Uncropped gels
a) Uncropped immunoblot gel images associated with Extended Data Fig. 2b. Representative crop shown in black box. b) Uncropped representative image and additional quantified western blots shown for Extended Data Fig. 2h & 2i. Details for western blot loading controls show below and at right of gels. c) Uncropped DNA amplicon gels associated with Extended Data Fig. 6b.

References:

1. Tsai, S. Q. *et al.* GUIDE-seq enables genome-wide profiling of off-target cleavage by CRISPR-Cas nucleases. *Nat Biotechnol* **33**, 187–197 (2015).
2. Smith, J. mugnierlab/Smith2026: Release for publication. Zenodo https://doi.org/10.5281/ZENODO.18716076 (2026).
3. Beaver, A. K. *et al.* Tissue spaces are reservoirs of antigenic diversity for Trypanosoma brucei. *Nature* **636**, 430–437 (2024).
4. Cross, G. A. M., Kim, H.-S. & Wickstead, B. Capturing the variant surface glycoprotein repertoire (the VSGnome) of Trypanosoma brucei Lister 427. *Molecular and Biochemical Parasitology* **195**, 59–73 (2014).
5. Amos, B. *et al.* VEuPathDB: the eukaryotic pathogen, vector and host bioinformatics resource center. *Nucleic Acids Research* **50**, D898–D911 (2022).
6. Alvarez-Jarreta, J. *et al.* VEuPathDB: the eukaryotic pathogen, vector and host bioinformatics resource center in 2023. *Nucleic Acids Research* **52**, D808–D816 (2024).
7. Müller, L. S. M. *et al.* Genome organization and DNA accessibility control antigenic variation in trypanosomes. *Nature* **563**, 121–125 (2018).
8. Rico, E., Jeacock, L., Kovářová, J. & Horn, D. Inducible high-efficiency CRISPR-Cas9-targeted gene editing and precision base editing in African trypanosomes. *Sci Rep* **8**, 7960 (2018).
9. Schulz, D., Mugnier, M. R., Boothroyd, C. E. & Papavasiliou, F. N. Detection of Trypanosoma brucei Variant Surface Glycoprotein Switching by Magnetic Activated Cell Sorting and Flow Cytometry. *JoVE* 54715 (2016) doi:10.3791/54715-v.
10. Rojas, F. *et al.* Oligopeptide Signaling through TbGPR89 Drives Trypanosome Quorum Sensing. *Cell* **176**, 306-317.e16 (2019).
11. Oldrieve, G. R. *et al.* Mechanisms of life cycle simplification in African trypanosomes. *Nat Commun* **15**, 10485 (2024).
12. Kim, H.-S., Li, Z., Boothroyd, C. & Cross, G. A. M. Strategies to construct null and conditional null Trypanosoma brucei mutants using Cre-recombinase and loxP. *Mol Biochem Parasitol* **191**, 16–19 (2013).
13. Barrett, B., LaCount, D. J. & Donelson, J. E. Trypanosoma brucei: a first-generation CRE-loxP site-specific recombination system. *Experimental Parasitology* **106**, 37–44 (2004).
14. Paterou, A. *et al.* A comprehensive toolkit for protein localization and functional analysis in trypanosomatids. *Open Biol* **15**, 240361 (2025).
15. Dean, S. *et al.* A toolkit enabling efficient, scalable and reproducible gene tagging in trypanosomatids. *Open Biol* **5**, 140197 (2015).
16. Schumann Burkard, G., Jutzi, P. & Roditi, I. Genome-wide RNAi screens in bloodstream form trypanosomes identify drug transporters. *Molecular and Biochemical Parasitology* **175**, 91–94 (2011).
17. Beneke, T. *et al.* A CRISPR Cas9 high-throughput genome editing toolkit for kinetoplastids. *R. Soc. open sci.* **4**, 170095 (2017).
18. Scahill, M. D., Pastar, I. & Cross, G. A. M. CRE recombinase-based positive–negative selection systems for genetic manipulation in Trypanosoma brucei. *Molecular and Biochemical Parasitology* **157**, 73–82 (2008).

19. Zheng, Z. *et al.* Anchored multiplex PCR for targeted next-generation sequencing. *Nat Med* **20**, 1479–1484 (2014).
20. Martin, M. Cutadapt removes adapter sequences from high-throughput sequencing reads. *EMB.journal* **17**,.
21. Fu, L., Niu, B., Zhu, Z., Wu, S. & Li, W. CD-HIT: accelerated for clustering the next-generation sequencing data. *Bioinformatics* **28**, 3150–3152 (2012).
22. Li, W. & Godzik, A. Cd-hit: a fast program for clustering and comparing large sets of protein or nucleotide sequences. *Bioinformatics* **22**, 1658–1659 (2006).
23. Monica Mugnier & Alexander Beaver. mugnierlab/VSGSeqPipeline: Release for Smith, et al 2026. Zenodo https://doi.org/10.5281/ZENODO.18716085 (2026).
24. Csardi, G. & Nepusz, T. The igraph software package for complex network research. *InterJournal* **1695**, (2006).
25. Csárdi, G. *et al.* igraph for R: R interface of the igraph library for graph theory and network analysis. Zenodo https://doi.org/10.5281/ZENODO.7682609 (2024).
26. Edgar, R. C. Search and clustering orders of magnitude faster than BLAST. *Bioinformatics* **26**, 2460–2461 (2010).
27. Li, H. Minimap2: pairwise alignment for nucleotide sequences. *Bioinformatics* **34**, 3094–3100 (2018).
28. Li, H. New strategies to improve minimap2 alignment accuracy. *Bioinformatics* **37**, 4572–4574 (2021).
29. Danecek, P. *et al.* Twelve years of SAMtools and BCFtools. *GigaScience* **10**, giab008 (2021).
30. Altschul, S. F., Gish, W., Miller, W., Myers, E. W. & Lipman, D. J. Basic local alignment search tool. *J Mol Biol* **215**, 403–410 (1990).
31. Kim, D., Paggi, J. M., Park, C., Bennett, C. & Salzberg, S. L. Graph-based genome alignment and genotyping with HISAT2 and HISAT-genotype. *Nat Biotechnol* **37**, 907–915 (2019).
32. Girasol, M. J. *et al.* Immunoprecipitation of RNA–DNA hybrid interacting proteins in *Trypanosoma brucei* reveals conserved and novel activities, including in the control of surface antigen expression needed for immune evasion by antigenic variation. *Nucleic Acids Research* **51**, 11123–11141 (2023).
33. MacGregor, P., Savill, N. J., Hall, D. & Matthews, K. R. Transmission Stages Dominate Trypanosome Within-Host Dynamics during Chronic Infections. *Cell Host & Microbe* **9**, 310–318 (2011).
