# Supplementary material for: DNA damage drives antigen diversification in Trypanosoma brucei
Source: Nature. 2026 Apr 8;654(8117):219–28. doi: 10.1038/s41586-026-10337-6 (PMC13233330; doi:10.1038/s41586-026-10337-6)
Supplement: Supplementary file 2 — Reporting Summary [file 41586_2026_10337_MOESM2_ESM.pdf]

nature portfolio

Corresponding author(s): Monica Mugnier

Last updated by author(s): Feb 19, 2026

Reporting Summary

Nature Portfolio wishes to improve the reproducibility of the work that we publish. This form provides structure for consistency and transparency in reporting. For further information on Nature Portfolio policies, see our Editorial Policies and the Editorial Policy Checklist.

Statistics

For all statistical analyses, confirm that the following items are present in the figure legend, table legend, main text, or Methods section.

n/a	Confirmed	
☐	☒	The exact sample size (*n*) for each experimental group/condition, given as a discrete number and unit of measurement
☐	☒	A statement on whether measurements were taken from distinct samples or whether the same sample was measured repeatedly
☐	☒	The statistical test(s) used AND whether they are one- or two-sided<br>*Only common tests should be described solely by name; describe more complex techniques in the Methods section.*
☐	☒	A description of all covariates tested
☐	☒	A description of any assumptions or corrections, such as tests of normality and adjustment for multiple comparisons
☐	☒	A full description of the statistical parameters including central tendency (e.g. means) or other basic estimates (e.g. regression coefficient) AND variation (e.g. standard deviation) or associated estimates of uncertainty (e.g. confidence intervals)
☐	☒	For null hypothesis testing, the test statistic (e.g. *F*, *t*, *r*) with confidence intervals, effect sizes, degrees of freedom and *P* value noted<br>*Give P values as exact values whenever suitable.*
☐	☒	For Bayesian analysis, information on the choice of priors and Markov chain Monte Carlo settings
☐	☒	For hierarchical and complex designs, identification of the appropriate level for tests and full reporting of outcomes
☒	☐	Estimates of effect sizes (e.g. Cohen's *d*, Pearson's *r*), indicating how they were calculated

Our web collection on statistics for biologists contains articles on many of the points above.

Software and code

Policy information about availability of computer code

Data collection	For VSG Seq, libraries were sequenced with 100bp single-end reads on a NovaSeq6000. For VSG AMP Seq, reads were sequenced with a MiSeq or NovaSeq6000 (muMT mouse samples) with custom index1 and read2 primers with paired-end reads using the following cycle conditions: "151|8|33|131" . Single parasite clones were sequenced by Plasmidsaurus using oxford nanopore technology and their custom analysis and annotation software. other parasite clones were sequenced with Sanger sequencing using custom primers reported in the manuscript.<br><br>Nanopore sequencing for VSG amplicons from muMT infected mouse blood (originally infected with Tb427VSG-8) was performed with a PromethION using a 10.4.1 flow cell and the SQK-LSK114 kit.
Data analysis	The VSG-Seq pipeline (1) is available on github: http://github.com/mugnierlab/VSGSeqPipeline The software versions used were: Trinity(2.8.5), Biopython(1.72), Blast(2.9), Bedtools(2.29.2), cd-hit(4.8.1), trim-galore(0.6.4), bowtie(v1.2.3), and samtools(1.9).<br><br>VSG AMP Seq pipeline was created for custom analysis of the VSG AMP Seq reads. The software is available on http://github.com/mugnierlab/Smith2026/tree/main/VSG-AMP-Seq. A module of VSG-AMP-seq was also deposited onto CodeOcean: 10.24433/CO.3005244.v1 and 10.24433/CO.3283350.v1. The software versions used were: Python(3.8.19), biopython(1.78), cutadapt(3.5), trim-galore(v0.6.4), pandas(1.2.1), python-levenshtein(0.25.1), progress-bar(2.5), regex(2.5.82), bowtie(1.3.1), cd-hit(v4.8.1). Modules are manually run via: index_addition, primer_sort, trim, demultiplex, consol_reads, vsg_align, define_read_consensus, identify_mosaics. The output of this can be run through custom R functions included in mosaic_graphing_functions. Analysis was run with R(4.0.2). Quantification of reads uses quant_R1s_bowtie. To run this pipeline custom VSG-specific files and experiment-specific are required including: global_target.py, a _primer.txt file, a _primers.fasta file, a fasta file containing the target sequence, and a custom barcodes file.

VSGnome analysis can be run with the pipeline genome_VSG_analysis. It requires blast(2.10.0). VSG structural prediction was performed by isolating N-termini with the following available scripts: github.com/mugnierlab/find_VSG_Ndomains. It requires software SignalP(6.0), HMMscan(3.1b2), and ColabFold(1.5.5). Visualization used UCSF ChimeraX (1.7.1).

Scripts to identify the in vivo VSG-8 sequences can be found at github.com/mugnierlab/smith2025/tree/main/altVSG8_nanopore_analysis. The software versions used were: Dorado (7.2.13), samtools (1.22.1), cutadapt (4.9), sed (4.5), hisat2 (2.1.0), and BLAST(2.16.0+).

Scripts to reconstruct mosaic VSGs from mixed nanopore populations can be found at github.com/mugnierlab/smith2026/tree/main/nanopore_colony_consensus_builder. The software versions used were: sed(4.5), cd-hit(4.8.1), minimap2(2.30-r1287), and samtools(1.22.1).

FlowJo(10.8.1) was used to perform gating and analyze flow cytometry data.

Western blots were analyzed with FIJI(2.14.0/1.54f). Code used to generate all figures is found at github.com/mugnierlab/Smith2026/tree/main/Figures.

For manuscripts utilizing custom algorithms or software that are central to the research but not yet described in published literature, software must be made available to editors and reviewers. We strongly encourage code deposition in a community repository (e.g. GitHub). See the Nature Portfolio guidelines for submitting code & software for further information.

Data

Policy information about availability of data

All manuscripts must include a data availability statement. This statement should provide the following information, where applicable:

- Accession codes, unique identifiers, or web links for publicly available datasets
- A description of any restrictions on data availability
- For clinical datasets or third party data, please ensure that the statement adheres to our policy

Data for generating figures, i.e. the output of VSG-AMP-seq, are available on github: https://github.com/mugnierlab/Smith2025/tree/main/Figures. The raw sequencing reads for all data are available in the National Center for Biotechnology Information (NCBI) Sequence Read Archive under accession number PRJNA1140873.

All mosaic VSGs isolated from individual clones, and donor VSG amplicon sequences with cooresponding mosaic VSG sequences are on github.com/mugnierlab/Smith2026.

VSG-seq from in vivo mouse infections (blood & tissues) is found in Beaver et al. and at github.com/mugnierlab/Beaver2022.

VSG sequences for analysis are found at tryps.rockefeller.edu/Sequences.html. Additional VSG sequences were identified in TriTrypDB release 66, Lister2018 genome.

VSG N-terminal domain HMM profile can be found at github.com/mugnierlab/find_VSG_Ndomains.

Research involving human participants, their data, or biological material

Policy information about studies with human participants or human data. See also policy information about sex, gender (identity/presentation), and sexual orientation and race, ethnicity and racism.

Reporting on sex and gender	n/a
Reporting on race, ethnicity, or other socially relevant groupings	n/a
Population characteristics	n/a
Recruitment	n/a
Ethics oversight	n/a

Note that full information on the approval of the study protocol must also be provided in the manuscript.

Field-specific reporting

Please select the one below that is the best fit for your research. If you are not sure, read the appropriate sections before making your selection.

☒ Life sciences ☐ Behavioural & social sciences ☐ Ecological, evolutionary & environmental sciences

For a reference copy of the document with all sections, see nature.com/documents/nr-reporting-summary-flat.pdf

Life sciences study design

All studies must disclose on these points even when the disclosure is negative.

Sample size

Based on our previous work, a sample size of 4-5 mice is typically sufficient for analyses of antigenic variation, so we sought to achieve this sample size for all experiments.
(Mugnier MR, Cross GA, Papavasiliou FN. The in vivo dynamics of antigenic variation in Trypanosoma brucei. Science. 2015 Mar 27;347(6229):1470-3. doi: 10.1126/science.aaa4502. PMID: 25814582; PMCID: PMC4514441
Beaver, A.K., Keneskhanova, Z., Cosentino, R.O. et al. Tissue spaces are reservoirs of antigenic diversity for Trypanosoma brucei. Nature 636, 430–437 (2024). https://doi.org/10.1038/s41586-024-08151-z)

muMT mice, as they are immunocompromised, sometimes died prematurely before they reached the second corresponding peak of parasitemia in the WT mice. We infected enough mice (15 of each mouse genotype) such that sufficient muMT mouse survival across all 3 infections could ensure sufficient sample size. Experiments resulted in n=7 muMT and n=7 WT mice, across 3 separate infections. For subsequent experiments with muMT mice, we infected 5 mice with parasites. Here when we infected mice with RAD51-knockout parasites, we ended up with n=4, but are appropriately tentative with our conclusions from the results. VSG-8 expressing infections in muMT mice were n=5 with some mice dying before D15, like in infections with AnTat1.1. We were able to collect samples from all of these mice, extending our time course over multiple time points. Sample size at each time point limits the statistical significance of the number of mosaic VSGs observed, however these infections were sufficient to identify numerous mosaic VSGs generated in vivo.

Data exclusions

We excluded additional WT mice which survived - such that the number of WT mice and the number of muMT mice matched. This was chosen randomly among the samples - across the 3 experiments.

Replication

These experiments represent 7 biological replicates of each genotype across 3 independent experiments. Each infection was started at a distinct time with a distinct vial of parasites. muMT and WT mice were infected within an experiment at the same time. RAD51-knockout and VSG-8 mouse experiments were performed once, but with 4-5 biological replicates, from one vial of parasites. No additional attempts were made to perform these experiments beyond what is described here.

Randomization

Mice were housed together based on mouse genotype. Mice were randomly numbered 1-5 and treated in that order throughout the experiment. muMT mice were treated and handled first to prevent secondary infections. Sample processing occurred in batches based on experiment, but included both genotypes.

Blinding

Blinding of the samples was not necessary for the investigators since each sample was processed through a pipeline which treated them all equivalently. In addition, all wildtype parasite samples were sequenced with both VSG-Seq and VSG-AMP-Seq where results matched.

Reporting for specific materials, systems and methods

We require information from authors about some types of materials, experimental systems and methods used in many studies. Here, indicate whether each material, system or method listed is relevant to your study. If you are not sure if a list item applies to your research, read the appropriate section before selecting a response.

Materials & experimental systems

n/a	Involved in the study	
☐	☒	Antibodies
☐	☒	Eukaryotic cell lines
☒	☐	Palaeontology and archaeology
☐	☒	Animals and other organisms
☒	☐	Clinical data
☒	☐	Dual use research of concern
☒	☐	Plants

Methods

n/a	Involved in the study	
☒	☐	ChIP-seq
☐	☒	Flow cytometry
☒	☐	MRI-based neuroimaging

Antibodies

Antibodies used

We used a custom AnTat1.1 polyclonal CRD-depleted (cross-reactive domain) antibody provided by Jay Bangs. Other antibodies used included: mouse anti FLAG (M2) monoclonal antibody (Millipore Sigma, F3165-1MG), mouse anti EF1a (CBP-KK1 clone) (Millipore 903 Sigma, 05-235), and rabbit anti g-H2A was a kind gift from Galadriel Hovel-Miner based upon Glover and Horn. We also generated mouse polyclonal antisera against AnTat1.1 expressing Lister427 parasites. We had 4 biological replicates, M1-M4. Secondary antibodies for western blot were: goat anti mouse (Cell Signaling, 7076S) or goat anti rabbit-HRP-conjugated secondary (Cell Signaling, 7074S). Secondary antibodies for flow cytometry were: Alexa Fluor 647-conjugated goat anti rabbit IgG (H+L), 911 F(ab')2 Fragment (Cell Signaling, 4414S) and goat anti mouse IgG (H+L), F(ab')2 Fragment (Cell Signaling, 4410S).

Validation

The AnTat1.1 polyclonal antibody has been validated in Beaver et al, 2024, but also used to stain AnTat1.1 expressing parasites and Lister427 parasites which express VSG-2. Positive Signal was only observed for the parasites expressing AnTat1.1. Live/dead propidium iodide staining was validated using heat killed parasites. g-H2A was detailed in Glover and Horn Ref: Glover, L. & Horn, D. Trypanosomal histone gH2A and the DNA damage response. Mol Biochem Parasitol. (2012)183(1):78-83. This was validated by a peptide competition assay. AnTat1.1 expressing Lister427 antisera from the mice was stained against intact AnTat1.1 expressing parasites and VSG-228 which is antigenically distinct. These are in figure 5.

No statements were made for mouse anti FLAG M2 on the manufacturer's website. Western blot signal is only observed in trypanosome lysate when recombinant FLAG-tag protein is introduced.
mouse anti EF1a is validated for use in IP and WB per the manufacturer. This clone was partially validated here:
Kaur KJ, Ruben L. Protein translation elongation factor-1 alpha from Trypanosoma brucei binds calmodulin. J Biol Chem. 1994 Sep 16;269(37):23045-50. PMID: 8083206.

The following publications have used CBP-KK1 as a loading control for Trypanosoma brucei western blots:
Keneskhanova, Z., McWilliam, K.R., Cosentino, R.O. et al. Genomic determinants of antigen expression hierarchy in African trypanosomes. Nature 642, 182–190 (2025). https://doi.org/10.1038/s41586-025-08720-w
Escrivani DO, Scheidt V, Tinti M, Faria J, Horn D. Competition among variants is predictable and contributes to the antigenic variation dynamics of African trypanosomes. PLoS Pathog. 2023 Jul 17;19(7):e1011530. doi: 10.1371/journal.ppat.1011530. PMID: 37459347; PMCID: PMC10374056.

Eukaryotic cell lines

Policy information about cell lines and Sex and Gender in Research

Cell line source(s)	EATRO 1125 AnTat1.1 90-13 T. brucei (gifted from Keith Mathews, Ref: Engstler, M. & Boshart, M. Cold shock and regulation of surface protein trafficking convey sensitization to inducers of stage differentiation in Trypanosoma brucei . Genes Dev. 18, 2798(2004))<br>Monomorphic Single Marker Lister427 VSG221 TetR T7RNAP bloodstream form (NR42011; Lot: 61775530) Wirtz, E. Leal, S., Ochatt, C. & Cross, G.A.M. A tightly regulated inducible expression system for conditional gene knock-outs and dominant negative genetics in Trypanosoma brucei. Mol. Biochem. Parasitol. 99, 89-101(1999)<br><br>Monomorphic Single Marker 427 1339 Cas9 TetR T7RNAP (bloodstream form) (NR-56793; LOT: 70056027)<br><br>EATRO1125 AnTat1.1 J1339 pleiomorphic parasites were a gift from Keith Matthews.
Authentication	Cell lines were authenticated via sequencing of the VSG expressed by the parasite and PCR of drug resistance markers and corresponding drug resistance.
Mycoplasma contamination	Cells lines were not confirmed for mycoplasma contamination
Commonly misidentified lines<br>(See ICLAC register)	No commonly misidentified lines were used.

Animals and other research organisms

Policy information about studies involving animals; ARRIVE guidelines recommended for reporting animal research, and Sex and Gender in Research

Laboratory animals	C57Bl/6J (WT, strain # 000664 Jackson Laboratory) or B6.129S2-Ighmtm1Cgn/J (µMT-, strain #002288 Jackson Laboratory) between 8-12 weeks were used for the study. Mouse were injected intravenously in the tail vein with 5 parasites or IP with 100 parasites for antibody generation.
Wild animals	No wild animals were used in this study.
Reporting on sex	Only female mice were used for this study. Previous experiments in the field suggest sex does not have a major influence on antigenic variation. Cost also limited our ability to perform this experiment in multiple sexes.
Field-collected samples	No field collected samples were used in this study.
Ethics oversight	All animal studies were approved by the Johns Hopkins Animal Care and Use Committee (protocol # MO22H163).

Note that full information on the approval of the study protocol must also be provided in the manuscript.

Plants

Seed stocks	*Report on the source of all seed stocks or other plant material used. If applicable, state the seed stock centre and catalogue number. If plant specimens were collected from the field, describe the collection location, date and sampling procedures.*
Novel plant genotypes	*Describe the methods by which all novel plant genotypes were produced. This includes those generated by transgenic approaches, gene editing, chemical/radiation-based mutagenesis and hybridization. For transgenic lines, describe the transformation method, the number of independent lines analyzed and the generation upon which experiments were performed. For gene-edited lines, describe the editor used, the endogenous sequence targeted for editing, the targeting guide RNA sequence (if applicable) and how the editor was applied.*
Authentication	*Describe any authentication procedures for each seed stock used or novel genotype generated. Describe any experiments used to assess the effect of a mutation and, where applicable, how potential secondary effects (e.g. second site T-DNA insertions, mosiacism, off-target gene editing) were examined.*

Flow Cytometry

Plots

Confirm that:

☒ The axis labels state the marker and fluorochrome used (e.g. CD4-FITC).

☒ The axis scales are clearly visible. Include numbers along axes only for bottom left plot of group (a 'group' is an analysis of identical markers).

☒ All plots are contour plots with outliers or pseudocolor plots.

☒ A numerical value for number of cells or percentage (with statistics) is provided.

Methodology

Sample preparation	In 96 well plates, 200,000 parasites were stained with 1:20,000 rabbit anti AnTat1.1 primary antibody (Jay Bangs) or 1:1000 mouse anti AnTat1.1 serum for ten minutes at 4C while shaking in PBS + 10mg/mL glucose. Parasites were washed once with 100uL PBS + glucose. Then, parasites were stained with Alexa Fluor 647-conjugated goat anti rabbit IgG (H+L), F(ab')2 Fragment (Cell Signaling, 4414S) or goat anti mouse IgG (H+L), F(ab')2 Fragment (Cell Signaling, 4410S) at 1:1000 at 4C while shaking in PBS + glucose. After washing again with 100uL PBS + glucose, parasites were resuspended in PBS + glucose + 1:20 Propidium Iodide (BD Biosciences, 556463) and analyzed on a Attune Nxt Flow cytometer (Invitrogen). Data analysis was performed using FlowJo v10.
Instrument	Attune Nxt Flow Cytometer (Invitrogen)
Software	FlowJo (10.6.1)
Cell population abundance	Each parasite was obtained from a single cell clone, in a limited dilution 96-well plate such that less than 30 clones were present. The VSG expressed by these parasites was sequenced, and purity of individual sequencing reads was assessed to ensure just one VSG was expressed by the clone and it had not arisen from two clones, or a subpopulation had undergone a switch. Parasites were counted before they were plated and a similar number of parasites was run through the instrument to assess global staining of the parasites.
Gating strategy	For flow cytometry experiments, we first gated on T. brucei cells using FSC-A vs SSC-A. Next, we gated on single cells using FSC-A vs FSC-H. Finally, we gated on alive cells using the PI negative staining using the compensated Alexa Fluor 647-A vs compensated PI-A. Alive cells were gated and analyzed in histograms in Figure 6.

☒ Tick this box to confirm that a figure exemplifying the gating strategy is provided in the Supplementary Information.
